# Peer- and community-led responses to HIV: A scoping review

**DOI:** 10.1371/journal.pone.0260555

**Published:** 2021-12-01

**Authors:** George Ayala, Laurel Sprague, L. Leigh-Ann van der Merwe, Ruth Morgan Thomas, Judy Chang, Sonya Arreola, Sara L. M. Davis, Aditia Taslim, Keith Mienies, Alessandra Nilo, Lillian Mworeko, Felicita Hikuam, Carlos Garcia de Leon Moreno, José Antonio Izazola-Licea

**Affiliations:** 1 MPact Global Action for Gay Men’s Health and Rights, Oakland, CA, United States of America; 2 Alameda County Department of Public Health, Oakland, CA, United States of America; 3 Joint United Nations Programme on HIV/AIDS (UNAIDS), Geneva, Switzerland; 4 Social, Health and Empowerment Feminist Collective of Transgender Women in Africa, East London, South Africa; 5 Innovative Response Globally to Transgender Women and HIV (IRGT), Oakland, CA, United States of America; 6 Global Network of Sex Work Projects, Edinburgh, Scotland; 7 International Network of People Who Use Drugs, London, United Kingdom; 8 Arreola Research, San Francisco, CA, United States of America; 9 Graduate Institute, Geneva, Switzerland; 10 Rumah Cemara, Bandung, Indonesia; 11 The Global Fund to Fight AIDS, Tuberculosis, and Malaria, Geneva, Switzerland; 12 Gestos–HIV, Communication and Gender, Recife, Brazil; 13 International Community of Women Living with HIV Eastern Africa, Kampala, Uganda; 14 AIDS and Rights Alliance for Southern Africa, Windhoek, Namibia; Médecins Sans Frontières (MSF), SOUTH AFRICA

## Abstract

**Introduction:**

In June 2021, United Nations (UN) Member States committed to ambitious targets for scaling up community-led responses by 2025 toward meeting the goals of ending the AIDS epidemic by 2030. These targets build on UN Member States 2016 commitments to ensure that 30% of HIV testing and treatment programmes are community-led by 2030. At its current pace, the world is not likely to meet these nor other global HIV targets, as evidenced by current epidemiologic trends. The COVID-19 pandemic threatens to further slow momentum made to date. The purpose of this paper is to review available evidence on the comparative advantages of community-led HIV responses that can better inform policy making towards getting the world back on track.

**Methods:**

We conducted a scoping review to gather available evidence on peer- and community-led HIV responses. Using UNAIDS’ definition of ‘community-led’ and following PRISMA guidelines, we searched peer-reviewed literature published from January 1982 through September 2020. We limited our search to articles reporting findings from randomized controlled trials as well as from quasi-experimental, prospective, pre/post-test evaluation, and cross-sectional study designs. The overall goals of this scoping review were to gather available evidence on community-led responses and their impact on HIV outcomes, and to identify key concepts that can be used to quickly inform policy, practice, and research.

**Findings:**

Our initial search yielded 279 records. After screening for relevance and conducting cross-validation, 48 articles were selected. Most studies took place in the global south (n = 27) and a third (n = 17) involved youth. Sixty-five percent of articles (n = 31) described the comparative advantage of peer- and community-led direct services, e.g., prevention and education (n = 23) testing, care, and treatment programs (n = 8). We identified more than 40 beneficial outcomes linked to a range of peer- and community-led HIV activities. They include improved HIV-related knowledge, attitudes, intentions, self-efficacy, risk behaviours, risk appraisals, health literacy, adherence, and viral suppression. Ten studies reported improvements in HIV service access, quality, linkage, utilization, and retention resulting from peer- or community-led programs or initiatives. Three studies reported structural level changes, including positive influences on clinic wait times, treatment stockouts, service coverage, and exclusionary practices.

**Conclusions and recommendations:**

Findings from our scoping review underscore the comparative advantage of peer- and community-led HIV responses. Specifically, the evidence from the published literature leads us to recommend, where possible, that prevention programs, especially those intended for people living with and disproportionately affected by HIV, be peer- and community-led. In addition, treatment services should strive to integrate specific peer- and community-led components informed by differentiated care models. Future research is needed and should focus on generating additional quantitative evidence on cost effectiveness and on the synergistic effects of bundling two or more peer- and community-led interventions.

## Introduction

Communities affected by health emergencies have a long history of acting to promote and protect the wellness and rights of their members, a fact that has been generally accepted in the public health field [1–3]. Communities are recognized as a ‘critical catalyst’ to achieving the health-related targets in Sustainable Development Goal (SDG) 3 [4]. Stakeholders at all levels in the HIV sector are also increasingly recognizing, with some resolve, that communities living with and disproportionately affected by HIV must now play an even more prominent role in the global response [5–8]. This recognition comes with the realization that the world is off-track to meet global HIV targets [9, 10] as evidenced by current epidemiologic trends in HIV incidence, prevalence, viral suppression, and AIDS deaths, especially among socially marginalized populations [11, 12]. Underlying these trends are persistent inequities in access to and funding for HIV prevention, care, and treatment, which are experienced by people living with HIV, young women and girls (especially in Sub-Saharan Africa), gay and bisexual men, people who use drugs, prisoners, sex workers, and transgender people (key and vulnerable populations) [13]. Unabated stigmatization, discrimination, violence, and criminalization directed at key and vulnerable populations fuel inequities, undermining traction made towards achieving global targets [14–25]. Over 60% of all new HIV infections worldwide are among key populations, which reflect said inequalities [9].

The COVID-19 pandemic and its aftermath further threaten the gains made in a global HIV response that is already off-track [26, 27]. People living with HIV are more likely than the general population to become severely ill with COVID-19 and more likely to die if hospitalized [28]. Investment in comprehensive HIV services, which is at present contracting [9], will likely shrink further as the world struggles to fund its response to the COVID-19 crisis. Moreover, key and vulnerable populations worldwide continue to be excluded from national social protection schemes, undermining critical and hard-fought gains in the fight against AIDS [29].

An international commitment to people-centred systems for health was enshrined in the United Nations (UN) 2021 Political Declaration on HIV and AIDS (“the Declaration”), building on strong commitments in the 2016 Political Declaration to ensure 30% of HIV service delivery would be community-led by 2030 [30]. In the 2021 Declaration, UN member States committed, as appropriate in the context of national programmes, to increase the proportion of HIV services delivered by community-led organizations to reach 30% of HIV testing and treatment services, 80% of HIV prevention services for high-risk populations, and 60% of programmes to achieve societally enabling environments by the year 2025 [31–33]

However, commitments made in 2016 have not yet translated into scaled-up coverage of community-led responses to HIV, despite donor recognition of the integral role communities can and do play [34]. The 2021 commitments are likely to see the same fate without concerted action. There are several reasons for this. First, the 2016 Declaration failed to clearly define what constitutes a ‘community-led’ programme, and until recently, the HIV sector had not come together to develop a shared definition of the term. As a result, activities led by people living with and disproportionately affected by HIV at the grassroots level have often been conflated with those led by national agencies or by international non-governmental organizations (INGOs), which may physically base themselves in communities, but that may not in fact have representatives from affected communities in senior management positions or on governance boards. This confusion over what legitimately constitutes a ‘community-led’ programme obfuscates responses at the local level, makes comparisons across studies challenging at best, and complicates monitoring, reporting, and analysis of progress towards commitments in the Declaration across regions.

Second, as previously mentioned, although there is recognition by governments, donors, and implementers of the need for community-led responses, the evidence to support them has lagged. This is because community-led organizations and networks, those with the greatest interest in documenting the effectiveness of their responses, seldom have the resources to conduct large-scale research [35]. Further, community-led studies might be critiqued as biased or conflicted or dismissed if experimental study designs, e.g., randomized control trials, which may be more appropriate for biomedical research, were not used to test for efficacy [36–39]. And because the HIV sector has been operating without a generally accepted definition, quantitatively measuring the comparative advantages of community-led responses is difficult to achieve.

Third, the global HIV response continues to operate with a “democratic deficit”. In other words, despite the expressed commitment to the Greater Involvement of People Living with AIDS (“the GIPA Principle”), a commitment which was explicitly mentioned for the first time in the 2021 Declaration, people living with and most affected by HIV are often not meaningfully and equitably engaged in decision-making, planning, financing, or implementing service delivery [32, 40–43]. As a result, funding intended for community-led organizations has sometimes been captured by programmes that in practice fail to consult or meaningfully partner with the communities they serve. What some authors have called the biomedicalization of the HIV response has further complicated decision-making regarding the various roles communities can and should play, including in service delivery [5].

Clarifying terminology and examining the evidence for greater coverage of community-led responses are of urgent importance. This article presents a definition for community-led responses developed in 2019 during a 2-day expert consultation convened by the Joint UN Programme on HIV/AIDS (UNAIDS) and endorsed by a diverse group of government and civil society experts in late 2020. We then present the results of a scoping review that examined and synthesized research focused on community- and peer-led HIV responses published in the past 40 years. Our aim is to strengthen the case for expanded coverage of community-led HIV responses, supported by a clear definition, peer-reviewed evidence, and a set of recommendations for decision makers and funders.

## Methods

### Community experts’ meeting to define ‘community-led’

Recognizing the challenges in monitoring progress towards the commitments in the Declaration and the need for a clearer definition of “community-led”, the UNAIDS Secretariat (JAI, LS) convened a 2-day consultation with community experts in June 2019, to suggest an operational definition of community-led responses to HIV, at the request of its Programme Coordinating Board (PCB). A subsequent consultation was planned to define ‘woman-led’, building from the definitions developed during the 2019 Expert Consultation in Montreux, Switzerland. The meeting was postponed because of the COVID-19 pandemic.

Experts who participated in the June 2019 consultation included representatives from the leading global transnational networks of people living with HIV and key populations, who together represent hundreds of national and regional community-led organizations. They included: the International Community of Women Living with HIV (ICW), Global Network of People Living with HIV (GNP+), Global Network of Young People Living with HIV (Y+), International Treatment Preparedness Coalition (ITPC), Global Network of Sex Work Projects (NSWP), International Network of People Who Use Drugs (INPUD), Innovative Response Globally to Transgender Women and HIV (IRGT), MPact Global Action for Gay Men’s Health and Rights, TB People (the network of people living with tuberculosis), Gestos–HIV, Communication and Gender, representatives from the NGO delegation to the UNAIDS PCB, and members of the Communities Delegation to the Global Fund to Fight AIDS, Tuberculosis, and Malaria (“the Global Fund”). Staff members from the Global Fund and the U.S. Centres for Disease Control and Prevention were also in attendance. The meeting participants recognised the priorities of people living with HIV, including women and young people living with HIV, gay men and bisexual men, people who use drugs, sex workers, and transgender people as an integral part of their consensus-building deliberations.

Experts began their meeting with a review of findings from a global survey undertaken by UNAIDS just prior to the consultation. The survey, offered in four languages (English, French, Spanish, and Russian), was designed to canvass diverse definitions for ‘community’ and to identify core elements of ‘community-led’ and ‘key population-led’ in the context of the HIV/TB response. There were 475 completed surveys from respondents, representing 97 countries. Experts also studied policy documents and discussed ways to use the definition to monitor support and funding for critical community-led programmes. The meeting resulted in working definitions for the terms “community-led organizations”, “community-led responses”, “key population-led organizations”, and “key populations-led responses”. Meeting participants defined community-led responses as:

*…actions and strategies that seek to improve the health and human rights of their constituencies, that are specifically informed and implemented by and for communities themselves and the organizations, groups, and networks that represent them*.

*Community-led responses are determined by and respond to the needs and aspirations of their constituents. Community-led responses include advocacy, campaigning and holding decision-makers to account; monitoring of policies, practices, and service delivery; participatory research; education and information sharing; service delivery; capacity building, and funding of community-led organizations, groups, and networks. Community-led responses can take place at global, regional, national, subnational, and grassroots levels, and can be implemented virtually or in person*.

*Not all responses that take place in communities are community-led*.

Subsequent to this expert consultation, the proposed definitions of community-led responses and community-led organizations were vetted with two multistakeholder working groups for further input, resulting in minor changes to wording [44, 45]. The careful distinctions made in the definitions, initially developed by community experts and further refined through the multistakeholder processes, are important and include a clear emphasis on the meaningful inclusion of people living with HIV, gay and bisexual men, people who use drugs, sex workers, and transgender people in designing, implementing, managing, and evaluating programmes. Similar distinctions have been made by other groups [46]. All four definitions are presented in Table 1. The definition of community-led responses presented here informed the inclusion/exclusion criterion used in the scoping review, which focused on identifying evidence for the impact of community-led programmes on HIV outcomes.

**Table 1:** Definitions resulting from the Montreux experts consultation, June 2019.

**Community-led organization**	Community-led organizations, groups, and networks, whether formally or informally organized, are entities for which the majority of governance, leadership, staff, spokespeople, membership and volunteers, reflect and represent the experiences, perspectives, and voices of their constituencies and who have transparent mechanisms of accountability to their constituencies.
	Community-led organizations, groups, and networks are self-determining and autonomous, and not influenced by government, commercial, or donor agendas.
	Not all community-based organizations are community led.
**Community-led response**	Community-led responses are actions and strategies that seek to improve the health and human rights of their constituencies, that are specifically informed and implemented by and for communities themselves and the organizations, groups, and networks that represent them.
	Community-led responses are determined by and respond to the needs and aspirations of their constituents. Community-led responses include advocacy, campaigning and holding decision-makers to account; monitoring of policies, practices, and service delivery; participatory research; education and information sharing; service delivery; capacity building, and funding of community-led organizations, groups, and networks. Community-led responses can take place at global, regional, national, subnational, and grassroots levels, and can be implemented virtually or in person.
	Not all responses that take place in communities are community led.
**Key population-led organization**	Key population-led organizations and networks are led by people living with HIV, female, male and transgender sex workers, gay men and other men who have sex with men, people who use drugs, and transgender people. Key populations share experiences of stigmatization, discrimination, criminalization, and violence and shoulder disproportionate HIV disease burden in all parts of the world.
	Key population-led organizations and networks are entities whose governance, leadership, staff, spokespeople, members, and volunteers reflect and represent the experiences, perspectives, and voices of their constituencies.
	Key population-led organizations and networks and their expertise are anchored in our lived experiences, which determine our priorities. We speak for ourselves and are an intrinsic part of the global HIV response.
**Key population-led response**	Key populations are primary actors in, and intrinsic to, the global HIV response. Our responses are transformational, based on our priorities, needs and rights. Key populations should be included, on our own terms and with consideration to varying social and structural determinants, at all levels of the global HIV response.
	Key population responses aim to strengthen the capacities of our communities and are committed to action, irrespective of resource availability. Key population communities are overlapping and thus our responses strive to be intersectional. Key populations choose our own representative and how we engage in HIV-, gender-, human rights-, and development-related processes.

### Scoping review

The scoping review began as a discussion between co-authors (GA, LS, JAI) and principal stakeholders involved in a technical consultation on social enablers as part of the UNAIDS-led 2025 Target Setting process. Building on this work, we conducted a literature search focused on research published between January 1982 (six months after the first cases of HIV/AIDS were published in the United States of America) and February 2021 [47]. The overall goals of this scoping study were to gather available evidence on community-led responses and their impact on HIV outcomes, and to identify key concepts that can be used to immediately inform policy, practice, and research [48]. We followed a five-step procedure that involved articulating a research question, identifying relevant studies, selecting studies, charting the data, and summarizing the findings [49–51]. Our study was guided by the question: *What evidence is there about the comparative advantages of community-led HIV responses*?

### Data sources and search strategy

The search was conducted on February 20, 21, and 22, 2021 by the lead author (GA) using PubMed/MEDLINE, Embase, and Web of Science. The search included articles published between January 1982-and February 2021. Due to resource limitations, we restricted the search to articles published in English and focused on HIV. We used a Boolean search strategy [52], which combined search terms as follows: “community led HIV” OR “peer led HIV” OR”community led AIDS” OR “peer led AIDS”.

### Screening

Only titles were reviewed for the first level of screening. Second-level screening involved review of abstracts to exclude articles not relevant to the search and to remove duplicates. Studies were eligible for inclusion if they described community-led responses to HIV and their outcomes. Understanding that a common definition for community-led was absent when many studies were published, we included the search term ‘peer-led’ and evaluated each article against the criteria described in the definition developed at the Montreux consultation. Our search strategy included randomized controlled trials, quasi-experimental, prospective, pre/post-test evaluation, and cross-sectional study designs. We excluded study protocols, feasibility studies, case studies, case reports, editorials, behavioural surveillance studies, biomedical or pharmaceutical studies, and program reports. We also excluded articles that were not HIV-related, and/or that did not describe a program or intervention that was community- or peer-led.

After review and removal of non-relevant articles and duplicates, the two lead authors (GA, LS) cross-validated selected records, with inter-rater agreement reached for 86% of retrieved publications. Inclusion and exclusion discrepancies were discussed and resolved. Full text articles were then retrieved for review after consensus was reached. All co-authors were invited to identify additional peer-reviewed articles and grey literature, which were added if they appeared relevant to the review and conformed to the inclusion criteria. The study characteristics from full articles were extracted and compiled into a single spreadsheet for additional validation and coding. Authors communicated via email to resolve any additional outstanding questions or disagreements. Simple descriptive statistics were calculated to summarize the characteristics of research and data [47]. Other than what we describe in the methods section of this paper, no formal review protocol exists.

## Results

### Search and selection of evidence

**Fig 1:**
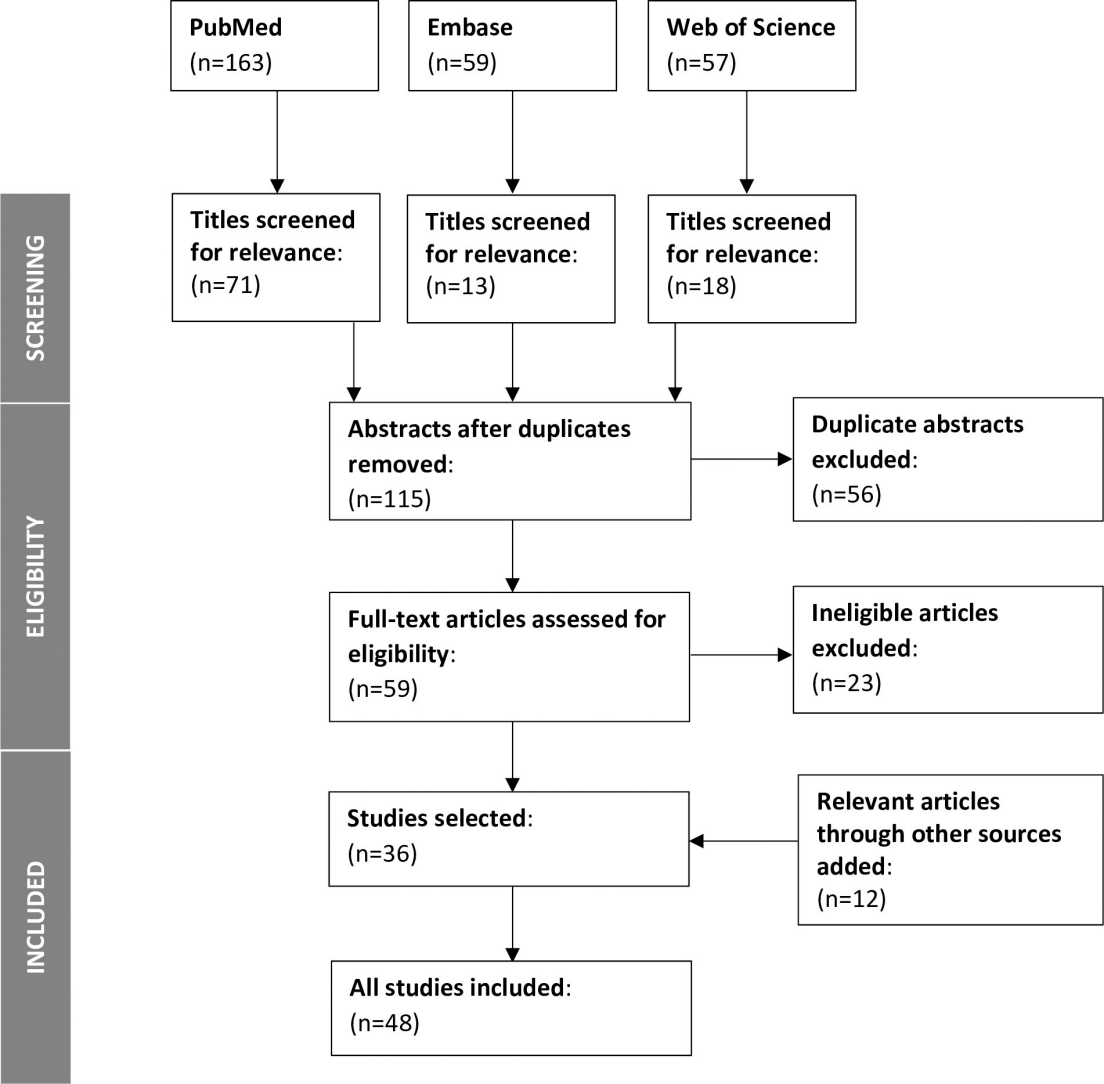
PRISMA flowchart of study selection process.

Our search yielded 279 potentially relevant records. After screening titles for relevance, 102 studies were excluded. After reviewing all abstracts remaining records for relevance, lead authors (GA, LS) then excluded another 56 articles. Sixty-two duplicate abstracts were identified and removed, leaving a total of 59 records. And after cross-validation and full text screening, 36 articles were selected. An additional 12 articles identified by co-authors and not captured by this scoping review were then added. The flow of articles in the selection process is presented in Fig 1.

Our search strategy yielded a total of 48 articles that met the inclusion criteria. Study methods and summary of findings are displayed in Table 2.

**Table 2:** Studies examining community- or peer-led service delivery and reported outcomes.

Year	Authors	Title	Method	Population	Intervention	Outcomes and Key Findings
1991	Shulkin JJ, et al.	Effects of a peer-led AIDS intervention with university students	Quasi-experimental	Youth	Prevention/ peer ed	Significant main effect for the intervention condition—mean change in scores (from pre-test to post-test) on knowledge, F(1, 81) = 20.0, p < 401, attitudes, F(1, 81) = 4.7, p = .033, and behavioural intentions, F(1, 81) = 24.2, p < .001.
1996	O’Hara P, et al	A peer-led AIDS prevention program for students in an alternative school.	Pre-post	Youth	Prevention/ peer ed	Over a three-month period, condom use at last intercourse increased among those who had sex from 44.8% to 55.2% (p < 0.025). At baseline, only 23.3% of students in the matched sample indicated they discussed HIV/AIDS with their peers, while at post-intervention, 67.7% of students indicated they had discussed HIV/AIDS with other students in their school (p < 0.001).
1996	Kegeles SM, et al.	The MPowerment Project: a community-level HIV prevention intervention for young gay men	Quasi-experimental	Gay/bi men—youth	Community empowerment	Two-tailed Wilcoxon matched-pair tests showed a decline in the frequency with which men reported unprotected anal intercourse with nonprimary partners in the intervention community (z = -2.35, P = .019, n = 97), but no significant change in the comparison community (z = -.45, P = .65, n = 85). There was also a decline in the frequency of unprotected anal intercourse with boyfriends in the intervention community (z = -1.72, P = .086, n = 17), but no significant change in the comparison community (z = -.84, P = .40, n = 9).
1996	Wingood GM, et al.	HIV sexual risk reduction interventions for women: a review	Lit review	Women	Prevention/ peer ed	Five RCTs (697 participants), 1 non-randomised trial (214 participants), and 1 before-and-after trial (241 participants) were included. All the theoretically based interventions (all investigated in RCTs) were effective in increasing condom use. The lengths of follow-up of these trials ranged from 3 to 12 months. All effective interventions emphasised gender-related influences on risk, were peer-led, and were multiple-session programmes.
1999	Kegeles SM, et al.	Mobilizing young gay and bisexual men for HIV prevention: a two-community study	Quasi-experimental	Gay/bi men—youth	Community empowerment	Sexual risk behaviour was stable between the two baseline assessments. From pre- to post-intervention, there were significant reductions in the proportions of young gay men reporting unprotected anal intercourse in the past 2 months with men in general, with boyfriends, and with non-primary partners. Analyses of unprotected anal sex with non-primary partners continued to decline after the intervention ended.
2000	Leonard L, et al.	HIV prevention among male clients of female sex workers in Kaolack, Senegal: Results of a peer education program	Prospective/ longitudinal	Men	Prevention/ peer ed	Significant increases in men’s HIV-related knowledge, previous use of condoms (from 30.4% to 53.5%), and consistent condom use with regular sex partners were documented over the study period, as were significant declines in perceived barriers to condom use. Women’s postintervention reports indicate that a greater proportion of clients (including, but not limited to transport workers) ’always’ agree to use condoms (p < .01) compared with baseline and that fewer men offer more money for unprotected sex (p < .01).
2001	Kocken P, et al.	Effects of peer-led AIDS education aimed at Turkish and Moroccan male immigrants in The Netherlands: A randomised controlled evaluation study	Pre-post	Immigrants—men	Prevention/ peer ed	Using multilevel logistic regression analysis, improvements were found on knowledge about human immunodeficiency virus (HIV) transmission (OR = 5.9 and 95% Cl: 2.3–15.3) and risk appraisal for HIV infection (OR = 2.9 and 95% Cl: 1.3–6.3).
*2002	Flowers P, et al.	Does bar-based, peer-led sexual health promotion have a community-level effect amongst gay men in Scotland?	Quasi-experimental	Gay/bi men	Prevention/ peer ed	The outcome measures were reported hepatitis B vaccination; HIV testing; unprotected anal intercourse (UAI) with casual partners; negotiated safety; and amongst men reporting UAI with a regular partner, the proportion who knew their own and their partner’s HIV status. Significant differences in sexual health behaviours were observed across locations and across time, but the only significant intervention effects were amongst men who had direct contact with the intervention, with higher uptake of hepatitis B vaccination and HIV testing. The intervention did not produce community-wide changes in sexual health behaviours.
2005	Borgia P, et al.	Is peer education the best approach for HIV prevention in schools? Findings from a randomized controlled trial	RCT	Youth	Prevention/ peer ed	Changes in sexual behaviours, knowledge, prevention skills, risk perception and attitudes were first evaluated within each intervention group. For both groups, significant improvements in skills, knowledge, attitudes, and risk perception were observed. The peer-led group showed a 6.7% (95% C.I. 1.9–11.5) scores greater improvement in knowledge, compared to the teacher-led group. In neither group were improvements observed in condom use or number of sexual partners.
2005	Wolitski RJ, et al.	Effects of a peer-led behavioural intervention to reduce HIV transmission and promote serostatus disclosure among HIV-seropositive gay and bisexual men	RCT	Gay/bi men	Prevention/ peer ed	Compared with the standard intervention, fewer men assigned to the enhanced intervention reported unprotected receptive anal intercourse with a negative or unknown-serostatus partner at 3 months (21% versus 26%, P < 0.05). The enhanced intervention was associated with only a limited reduction in transmission risk at 3 months relative to the standard intervention.
*2007	Simoni JM, et al.	A randomized controlled trial of a peer support intervention targeting antiretroviral medication adherence and depressive symptomatology in HIV-positive men and women	RCT	People living with HIV	Community groups/ clubs/support	Intent-to-treat and as-treated analyses indicated no between-conditions intervention effects on the primary outcome of HIV-1 RNA viral load or any of the secondary outcomes at immediate postintervention or follow-up. Post hoc analyses within the intervention condition indicated greater intervention exposure was associated with higher self-reported adherence, higher social support, and lower depressive symptomatology at follow-up, even after controlling for baseline adherence.
2008	Reza-Paul S, et al.	Declines in risk behaviour and sexually transmitted infection prevalence following a community-led HIV preventive intervention among female sex workers in Mysore, India	Cross sectional	Sex workers	Mobilization/ advocacy/ monitoring	Increases in condom use were seen between baseline and follow-up surveys: condom use at last sex with occasional clients was 65% versus 90%, P< 0001; with repeat clients 53% versus 66%, P<0.001; and with regular partners 7% versus 30%, P<0.001. STI prevalence declined from baseline to follow-up: syphilis 25% versus 12%, P< 0.001; trichomonas infection 33% versus 14%, P< 0.001; chlamydial infection 11% versus 5%, P = 0.001; gonorrhoea 5% versus 2%, P = 0.03. HIV prevalence remained stable (26% versus 24%), and detuned assay testing suggested a decline in recent HIV infections.
2008	Sifunda S, et al.	The effectiveness of a peer-led HIV/AIDS and STI health education intervention for prison inmates in South Africa	Quasi-experimental	Incarcerated	Prevention/ peer ed	Significant interaction effects between intervention and prison on the measures of knowledge, F(2, 224) = 4.32,p < .05, and intention, F(2, 221) = 4.63,p < .05 –were observed. Simple effect analyses on knowledge showed that the effect of intervention was significant in the KZN2 prison, F(1, 228) = 13.25,p < .001, and MP2 prison F(1, 228) = 5.64,p < .05, with participants in the experimental group showing more knowledge than participants in the control group. As predicted, the intervention group agreed more with statements supporting communication about sex with future partners (M = 4.68, SD = 0.61) than the control group (M = 4.34,SD = 0.84).
2009	Hong, H, et al.	Long-term follow-up of a peer-led HIV/AIDS prevention program for married women in rural China	RCT	Youth	Prevention/ peer ed	In the intervention group, the knowledge score of reproductive health, HIV/AIDS and sexually transmitted disease rose from 21.66 to 31.72 one month later (P < 0.001). After one year it was still 30.97, and there was no significant difference between one month and one year (P > 0.05). After both the one month and one-year follow-up intervention, investigators found that more students declared that they would use condoms during sexual intercourse when compared with the control group (P < 0.001). No change was seen in either knowledge or behaviour intention in the control group.
*2010	Webel AR.	Testing a peer-based symptom management intervention for women living with HIV/AIDS	RCT	Women	Adherence	Mixed-effects regression indicated no significant difference between groups across time in total symptom intensity score and medication adherence. There was a significant difference between groups across time for two of the nine quality of life scales—HIV Mastery (chi(2) = 25.08; p<0.005) and Disclosure Worries (chi(2) = 24.67; p<0.005).
2011	Nglazi MD, et al.	Changes in programmatic outcomes during 7 years of scale-up at a community-based antiretroviral treatment service in South Africa	Prospective/ longitudinal	People living with HIV	Testing/care/ treatment	Viral suppression was observed, with ≥93% of patients having suppression <400 copies/mL at 16 weeks. Rates did not vary significantly between successive years of recruitment, indicative of adherence to treatment.
*2012	Michielsen K, et al.	Effectiveness of a peer-led HIV prevention intervention in secondary schools in Rwanda: results from a non-randomized controlled trial.	Prospective/ longitudinal	Youth	Prevention/ peer ed	Time trends in sexual risk behaviour (being sexually active, sex in last six months, condom use at last sex) were not significantly different in students from intervention and control schools, nor was the intervention associated with increased knowledge, perceived severity, or perceived susceptibility. It did significantly reduce reported stigmatization. Investigators identified several reasons for the observed limited effectiveness of peer education: 1) intervention activities (spreading information) were not tuned to objectives (changing behaviour); 2) young people preferred receiving HIV information from other sources than peers; 3) outcome indicators were not adequate and the context in which sex occurs was ignored.
2012	Saad A, et al.	An HIV-STI risk reduction program among undergraduate students at a northern Nigerian university: A randomized controlled field trial	RCT	Youth	Prevention/ peer ed	Respondents in the intervention arm showed significant improvements in knowledge about HIV and STIs, and in sexual risk behaviours and attitudes towards HIV-STI prevention. Conversely, there was no difference in tolerance toward people living with HIV assessed using the stigma scale. There were significant main effects for group (F0 155.94, p≤0.001, η(2) = 0.401); time (F0248.35, p≤0.001, η(2) = 0.516), and group × time interaction (F0162.96, p≤ 0.001, η(2) = 0.412) for HIV-related knowledge. Similarly, the main effects for group, time, and group × time interaction for STI knowledge, sexual risk behaviours, and attitudes were also significant.
2012	Baghianimoghadam MH, et al.	Peer-led versus teacher-led AIDS education for female high-school students in Yazd, Islamic Republic of Iran	Quasi-experimental	Youth—young women	Prevention/ peer ed	Post-intervention, the mean knowledge scores of the peer-led group increased more than 2-fold, from 15.9 (SD 4.4) to 33.7 (SD 1.9) out of 34). The knowledge scores of the control group remained the same over 2 months [15.6 (SD 5.0) versus 15.8 (SD 4.9)]. After the intervention there was a highly significant increase in the peer led group in mean scores on knowledge (p< 0.001) and all constructs of the health belief model (p< 0.001).
2012	Ibrahim N, et al.	Effectiveness of peer-led education on knowledge, attitude, and risk behaviour practices related to HIV among students at a Malaysian public university-a randomized controlled trial	RCT	Youth	Prevention/ peer ed	Significant improvements in knowledge in the intervention group as compared to the control group (Odds ratio, 1.75; 95% CI 1.01, 3.00; p = 0.04) and in attitudes related to HIV (Odds ratio 2.22; 95% CI 1.37, 3.61; p = 0.01). The odds of high-risk behaviour were significantly reduced in the intervention group as compared to the control group (Odds ratio 0.07; 95% CI 0.02, 0.34; p = 0.01).
2012	Xiao Z, et al.	HIV/sexual risk reduction interventions in China: a meta-analysis	Lit review	Mixed	Prevention/ peer ed	Twenty-six intervention studies. The reviewed interventions were successful in improving HIV knowledge (d = 0.706), condom use knowledge (d = 0.620), attitudes toward people living with HIV/AIDS (PLWHA; d = 0.625) and in increasing condom use with regular partners (d = 0.477), condom use with casual partners (d = 0.444), general condom use (d = 0.408), and condom use self-efficacy (d = 0.584) among target audiences. In addition, moderating analyses on three most examined variables, demonstrated that interventions that were peer-led were more likely to report a positive impact on condom use behaviour (p<0.001), HIV knowledge (p<0.001), or attitudes toward PLWHA (p<0.001).
2012	Aramburu MG, et al.	Educational impact of peer-intervention on the knowledge and attitudes about HIV/AIDS in adolescents in Panama.	Quasi-experimental	Youth	Prevention/ peer ed	improvement in knowledge and attitudes was observed in both the private (ES = 0.63) and the public (ES = 0.52) schools with the intervention. The idea of abstinence as disease prevention for high school students rose from 7% to 60% (public school) and from 27% to 62% (private school). Both schools receiving the intervention scored higher than their respective control groups (p < 0.001). The effect size for the private schools was ES = 0.63 (+ 21 percentile points) and for the public schools ES = 0.52 (+ 18 percentile points).
*2013	Thato R, et al.	A Brief, Peer-Led HIV Prevention Program for College Students in Bangkok, Thailand	Quasi-experimental	Youth	Prevention/ peer ed	Brief, peer-led HIV prevention program significantly increased knowledge of preventive behaviours (β = 2.67, P < .000), motivated participants to have a better attitude toward preventive behaviours (β = -5.26, P < .000), better subjective norms (β = -1.54, P < .000), and greater intentions to practice preventive behaviour (β = -1.38, P < .000). The program also significantly decreased perceived difficulty of AIDS/STIs preventive behaviours (β = 2.38, P < .000) and increased perceived effectiveness at AIDS/STIs preventive behaviour (β = -3.03, P < .000). However, it did not significantly increase AIDS/STIs preventive behaviours (β = 2.13, P > .05).
2013	Traore IT, et al.	Effect of a tailored intervention package on HIV-1 acquisition among young female sex workers in Ouagadougou, Burkina Faso	Prospective/ longitudinal	Sex workers	Prevention/ peer ed	The intervention used a tailored prevention-and-care integrated approach, with repeated peer-led HIV/STI education sessions, condoms provision, and medical care. 86% of participants completed 12-months follow-up and no woman seroconverted for HIV-1 (0/405 person-years, 95% CI: 0–0.03). The expected HIV-1 incidence in this group was 1.23/100 person-years (95% CI: 1.02–1.46). The mean number of regular partners decreased during the intervention (from 2 to 1, p < 0.001). Adjusted consistent condom use remained consistently very high with clients between 97% and 99%) and did not increase with regular partners (from 64% to 62%). The incidence of HSV-2 was 11/100 person-years (95% CI: 7–15), and the pregnancy rate was 28/100 person-years (95% CI: 23–32).
2013	Canadian Agency for Drugs and Technology in Health	Peer Support for Diabetes, Heart Disease and HIV/AIDS: A Review of the Clinical Effectiveness, Cost-effectiveness, and Guidelines.	Lit review	Gay/bi men	Community groups/ clubs/support	HIV-related component included 1 previous systematic review, 117 studies, (Simoni JM, et al, 2011) and two randomized control trials (Horvath KJ, et al. 2012 –adherence study–and Van Tamm et al, 2013 –Quality of Life study). Self-reported improvements across each outcome were observed. Gay/bisexual men in the intervention arm (M[difference score] = 8.3, SD = 32.6) reported significantly greater ART adherence compared to those in the control arm (M[difference score] = −3.7, SD = 27.2; t[105] = 2.06, p = .04, Cohen’s d = .40). Scores on overall Quality of Life measure improved at twelve months (Control—75.36 (9.6) vs Intervention—78.69 (8.47) at 12-months, p = 0.023).
2013	Suthar AB, et al.	Towards universal voluntary HIV testing and counselling: a systematic review and meta-analysis of community-based approaches.	Lit review	Gen pop	Testing/care/ treatment	One-hundred and seventeen studies (n = 864,651). Community-based and -led testing increased uptake (RR = 10.65, 95% CI 6.27–18.08), the proportion of first-time testers (RR = 1.23, 95% CI 1.06–1.42), the proportion of participants with CD4 counts above 350 (RR = 1.42, 95% CI 1.16–1.74), and obtained a lower positivity rate (RR 0.59, 95% CI 0.37–0.96), relative to facility-based approaches. This systematic review found that community-based HIV testing and counselling (HTC) reached populations earlier during HIV infection than facility-based HTC.
2014	Calloway, DS, et al.	Reducing the Risk of HIV/AIDS in African American College Students: An Exploratory Investigation of the Efficacy of a Peer Educator Approach	Pre-post	Youth	Prevention/ peer ed	There were significant differences between the mean scores of the control and intervention groups on HIV/AIDS general knowledge (t(77) = −3.71, p < .001) and HIV/AIDS prevention self-efficacy scores (t(75) = −1.96, p = .05) at the conclusion of the intervention. In addition, the mean difference between pre- and post-assessment HIV/AIDS prevention self-efficacy scores among the intervention group was statistically significant (M = −2.207, SD = 2.637, N = 28, p = .001).
*2014	Ye S, et al	Efficacy of peer-led interventions to reduce unprotected anal intercourse among men who have sex with men: A meta-analysis	Lit review-meta-analysis	Gay/bi men	Prevention/ peer ed	Twenty-two studies selected. Peer-led interventions reduced UAI with any sexual partners in meta-analysis (mean ES: -0.27; 95% confidence interval [CI]: -0.41, -0.13; P<0.01). Subgroup analyses demonstrated a statistically significant reduction of UAI in quasi-experimental studies (mean ES: 2 0.30; 95% CI: -0.50, -0.09; P = 0.01) and serial cross-sectional intervention studies (mean ES: -0.33; 95% CI: -0.57, -0.09; P = 0.01), but no significant reduction found in RCTs (mean ES: -0.15; 95% CI: -0.36, 0.07; P = 0.18) nor pre- and post-intervention studies (mean ES: -0.29; 95% CI: -0.69, 0.11; P = 0.15). Heterogeneity was large across these 15 studies (I(2) = 77.5%; P, 0.01), largely due to design differences in pre-and-post intervention studies and serial cross-sectional intervention studies. Peer-led HIV prevention interventions reduced the overall UAI, but the efficacy varied by study design.
2014	Jain B, et al.	Effect of peer-led outreach activities on injecting risk behaviour among male drug users in Haryana, India	National program monitoring	People who use drugs	Prevention/ peer ed/ outreach	The proportion of IDUs who shared needles substantially decreased from 2009 to 2011, particularly among those who attended three or more peer-led education sessions (49% vs 11%, p < 0.001) in a month. Further, subgroup analysis by frequency of injecting drugs demonstrated that this decline was significant among IDUs who injected frequently (adjusted odds ratio = 0.6, 95% confidence interval = 0.3–0.9, p = 0.043). Repeated peer-led outreach sessions are more effective than exposure to a single education session.
2014	Yan H, et al.	A peer-led, community-based rapid HIV testing intervention among untested men who have sex with men in China: an operational model for expansion of HIV testing and linkage to care	Quasi-experimental	Gay/bi men	Testing/care/ treatment	Compared with those in the surveillance surveys, men who have sex with men tested by the community-led organization were significantly more likely to be younger, single, non-resident of the province, more educated and used condoms less frequently. Higher proportions of HIV-positive men screened by the CBO received their confirmatory test results (98.1% vs 72.6%, p<0.001) and were linked to care (90.4% vs 42.0%, p<0.001). Trained peers providing rapid HIV testing with social support and case management through the early period following diagnosis can expand HIV testing and improve linkage to care.
2015	Kim SR, et al	Uptake of a women-only, sex-work-specific drop-in centre and links with sexual and reproductive health care for sex workers	Prospective/ longitudinal	Sex workers—women	Drop-in centre	Of 547 female sex workers included in the present analysis, 330 (60.3%) utilized the services during the 3-year study period. Service use was independently associated with age (adjusted odds ratio [AOR] 1.04; 95% confidence interval [CI] 1.03–1.06), Aboriginal ancestry (AOR 2.18; 95% CI 1.61–2.95), injection drug use (AOR 1.67; 95% CI 1.29–2.17), exchange of sex for drugs (AOR 1.40; 95%CI 1.15–1.71) and accessing sexual and reproductive health services (AOR 1.65; 95% CI 1.35–2.02). A sex-work-specific drop-in space for marginalized sex workers had high uptake. Women-centred and low-threshold drop-in services can effectively link marginalized women with services.
2015	Kerrigan D, et al.	A community empowerment approach to the HIV response among sex workers: effectiveness, challenges, and considerations for implementation and scale-up	Lit review	Sex workers	Community empowerment	Twenty-two studies (n = 30,325). Community empowerment was associated with reductions in HIV (odds ratio [OR]: 0.68; 95% confidence interval [CI]: 0.52–0.89), gonorrhoea (OR: 0.61; 95% CI: 0.46, 0.82), chlamydia (OR: 0.74; 95% CI: 0.57, 0.98), and high-titre syphilis (OR: 0.53; 95% CI: 0.41, 0.69) and increased consistent condom use with clients (OR: 3.27; 95% CI: 2.32, 4.62).
2016	Timol F, et al.	Addressing adolescents’ risk and protective factors related to risky behaviours: Findings from a school-based peer-education evaluation in the Western Cape	Quasi-experimental	Youth	Prevention/ peer ed	ANOVA for the intervention schools at time 0 (baseline) and time 1 (immediately post intervention) indicate significantly higher means for future orientation (3.840, p < .05), self-efficacy in sexual relations (9.173, p < .05), knowledge regarding HIV transmission (16.691, p < .01), knowledge regarding HIV prevention (6.423, p < .01) and knowledge in terms of a healthy relationship (6.6261, p < .05) compared to the baseline for the intervention schools. The ANOVA conducted for the intervention group at time 0 (baseline) and time 2 (delayed post intervention) shows a significantly higher mean for self-efficacy in sexual relationships (26.31, p < .05) and HIV knowledge (35.11, p < .05).
2016	Argento E, et al.	Social cohesion among sex workers and client condom refusal in a Canadian setting: implications for structural and community-led interventions.	Prospective/ longitudinal	Sex workers	Community mobilization	Longitudinal (n = 692, 1,681 observations). Higher levels of perceived social cohesion among sex workers retained a direct and independent effect on reduced client condom refusal [adjusted odds ratio (aOR) 0.97 per unit increase in social cohesion score, 95% CI 0.95 to 0.99], after adjusting for place of soliciting clients and age.
*2016	Nachega JB, et al	Community-based interventions to improve and sustain antiretroviral therapy adherence, retention in HIV care and clinical outcomes in low- and middle-income countries for achieving the UNAIDS 90-90-90 targets.	Lit review	People living with HIV	Testing/care/ treatment	Twenty-two studies– 11 randomized control trials (n = 5,861) and 11 cohort studies (n = 89,388). No statistical difference in ART adherence, virologic suppression, mortality, and loss to follow-up when the analysis was restricted to RCTs. In the pooled analysis from both RCTs and cohort studies, participants assigned to community-led ART had significantly higher rates of retention in care than those in facility-based ART at the end of the follow-up (80.3% vs. 75.9%—RR = 1.03, 95% CI 1.01 to 1.06, I2 = 0%). Participants assigned to community-led ART had statistically significant higher rates of treatment engagement than those in facility-based ART at the end of the follow-up period (89.4% vs. 84.9%—RR = 1.09, 95% CI 1.03 t0 1.15, I2 = 69%).
2016	Ayala G, et al.	Will the global HIV response fail gay and bisexual men and other men who have sex with men?	Cross-sectional, observjational	Gay/bi men	Testing/care/ treatment	In the multivariable analyses, participants (n = 4859) who reported higher levels of engagement with the gay community were significantly more likely to have had an HIV test and received the result (adjusted odds ratio (aOR) = 1.67, confidence interval (CI) = 1.38 to 2.03); to have participated in HIV prevention programmes three or more times in the past six months (if HIV negative) (aOR = 3.35, CI = 2.36 to 4.75); and to have reported ever using PrEP (aOR = 2.7, CI = 2.0 to 3.5). Participants who reported higher levels of engagement with the gay community were significantly more likely to be retained in care (among men living with HIV) (aOR = 2.46, CI = 1.22 to 4.95). The odds of being tested for HIV within the past 12 months (among those who had ever been tested) (aOR = 1.63, CI = 1.20 to 2.22) and participating in HIV prevention programmes (aOR = 19.89, CI = 13.42 to 29.49) were considerably higher for study participants who accessed these services from community-based organizations specifically focused on LGBT people.
2017	Shangani S, et al.	Effectiveness of peer-led interventions to increase HIV testing among men who have sex with men: a systematic review and meta-analysis	Lit review	Gay/bi men	Testing/care/ treatment	Seven studies (n = 6205) selected, including 2 quasi-experimental studies, 4 non-randomized pre- and-post intervention studies, and 1 cluster randomized trial. Four studies were from high-income countries, two were from Asia and only one from sub-Saharan Africa. Meta-analysis found HIV testing rates were statistically significantly higher in the peer-led intervention groups versus control groups (pooled OR 2.00, 95% CI 1.74–2.31). Among randomized trials, HIV testing rates were significantly higher in the peer-led intervention versus control groups (pooled OR: 2.48, 95% CI 1.99–3.08). Among the non-randomized pre- and post-intervention studies, the overall pooled OR for intervention versus control groups was 1.71 (95% CI 1.42–2.06), with substantial heterogeneity among studies (I2 = 70%, p < 0.02).
2018	Mantsios A, et al.	Community Savings Groups, Financial Security, and HIV Risk Among Female Sex Workers in Iringa, Tanzania	Cross sectional	Sex workers	Community groups/ clubs/support	Multivariable regression results indicated that participating in a savings group was significantly associated with nearly two times greater odds of consistent condom use with new clients in the last 30 days (aOR = 1.77, 95% CI 1.10–2.86). Exploratory mediation analysis indicated that the relationship between savings group participation and consistent condom use was partially mediated by financial security, as measured by monthly income.
2019	Indravudh PP, et al.	Community-led delivery of HIV self-testing targeting adolescents and men in rural Malawi: A cluster-randomised trial	RCT	Gen pop	Testing/care/ treatment	Community-led HIV self-testing following participatory workshops and brief didactic training achieved high HIV self-testing uptake, reaching more adolescents, men, older adults, and couples. Post-intervention surveys showed 74.4% of HIV self-testing arm participants reporting self-testing, with 2.3% testing positive and 0.39% pressured to self-test. Lifetime testing in adolescents was 84.6% versus 67.1% in self-testing and standard of care arm (adjusted risk ratio (aRR) 1.25, 95%CI 1.10 to 1.43), with differences greatest for younger ages and males. A higher proportion of males reported recent testing in the self-testing arm than standard of care (74.5% versus 33.9%, aRR 2.21, 95%CI 1.92 to 2.55), with similar effects among older adults (74.2% versus 31.6%, aRR 2.37, 95%CI 2.00 to 2.80). Knowledge of status within couples was higher in the self-testing than standard of care arm (71.3% versus 56.9%, aRR 1.24, 95%CI 1.08 to 1.42), but prevention knowledge did not differ.
2019	Naserirad M, et al.	Effectiveness of a peer-led HIV/AIDS education program on HIV-related health literacy of jailed adolescents in Tunis, Tunisia	Quasi-experimental	Incarcerated—youth	Prevention/ peer ed	When changes over time, from baseline to follow-up, were compared between the intervention and comparison groups, differences were found for HIV-related health literacy (p = 0.029), knowledge (p = 0.031), risk perception (p = 0.043), preventive self-efficacy (p = 0.031) and behavioural intention (p = 0.019). Peer-led HIV/AIDS education program contributes to the development of HIV-related health literacy of jailed adolescents.
2019	Fox M, et. al.	Adherence clubs and decentralized medication delivery to support patient retention and sustained viral suppression in care: Results from a cluster-randomized evaluation of differentiated ART delivery models in South Africa.	Quasi-experimental	People living with HIV	Adherence	Patients participating in adherence clubs had higher 1-year retention (89.5% vs 81.6%, aRD: 8.3%; 95% CI 1.1% to 15.6%) and comparable sustained 1-year viral suppression (80.0% vs 79.6%, aRD: 3.83.8%; 95% CI: -6.9% to 14.4%). Retention associations were stronger for men than women (men RD: 13.1%, 95% CI: 0.3% to 23.5%; women RD: 6.0%, 95% CI: −0.9% to 12.9%). With decentralized medication delivery, patients had lower retention (81.5% versus 87.2%, aRD: −5.9%; 95% CI: −12.5% to 0.8%) and comparable viral suppression versus standard of care (77.2% versus 74.3%, aRD: −1.0%; 95% CI: −12.2% to 10.1%). Investigators noted increased viral suppression among men (RD: 11.1%; 95% CI: −3.4% to 25.5%).
2019	Stangl AL, et al.	A systematic review of selected human rights programs to improve HIV-related outcomes from 2003 to 2015: What do we know?	Lit review	Key populations	Mobilization/ advocacy/ human rights	Twenty-three studies selected. Most community-led interventions sought to influence availability and accessibility of services. Most (83%) studies reported improvements in HIV-related health outcomes (i.e., knowledge of harm reduction programs, ever tested for HIV, number of sex partners, condom use, HIV transmission rate, HIV and STI incidence, access to and utilization of HIV prevention and treatment services). All five socio-ecological levels of influence were addressed. Most interventions addressed 2 or more of the 5 UNAIDS’ human rights programs.
2019	Strömdahl S, et al.	Uptake of peer-led venue-based HIV testing sites in Sweden aimed at men who have sex with men (MSM) and trans persons: a cross-sectional survey	Evaluation, cross-sectional	Gay/bi men	Testing/care/ treatment	This study evaluated the Testpoint project, the first large-scale programme in Sweden providing venue-based HIV testing by peer non-healthcare personnel for men who have sex with men and transgender people. Data suggest that the programme enabled first time testers to come, as well as promoted repeat testing among high-risk individuals. Five persons, 0.8% (95% CI 0.3 to 2.0) of the participants, tested positive for HIV. Four of them did not already know their HIV status. The HIV prevalence among those tested at Testpoint is higher than the estimated prevalence of 0.07% in the general population but lower than the prevalence estimates of 2%–6% among men who have sex with men in Sweden. The programme was especially successful in reaching foreign-born men, which constituted 55% of the participants. One-fifth of the study participants had never had an HIV test. One-fifth stated that they would not have tested at a healthcare facility.
2020	Baptiste S, et al.	Community-Led Monitoring: When Community Data Drives Implementation Strategies	Lit review	People living with HIV	Mobilization/ advocacy/ monitoring	Twelve studies, 4 monitoring models examined (health facility committees, citizen report cards, community score cards, Community treatment/health observatories). Community-led monitoring resulted in increased access and utilization of services, improved health, decreased mortality, reduced waiting times, improved community relationships, earlier initiation of antiretroviral treatment, infrastructure upgrades, reduced stockouts, increased HIV testing, and increased use of viral load testing in treatment monitoring. Within 18 months of the community treatment observatory implementation, there was an 8.4% decrease in ART stockouts and a 10.7% decrease in lab reagent stockouts for viral load testing. Over the same period, community observatory implementation resulted in 23,618 more people initiated on ART, 16,844 more viral load tests per-formed, a 29% increase in viral suppression rates, and an increased average quality of care rating (from 3.8 to 4.2 out of 5) across all monitored health sites.
*2020	Boucher LM, et al.	Peer-led self-management interventions and adherence to antiretroviral therapy among people living with HIV: A systematic seview	Lit review	People living with HIV	Adherence	Thirteen studies selected. Findings demonstrate unclear effectiveness for peer-led self-management interventions improving ART adherence. Evidence was limited with only seven studies measuring this outcome and some risk of bias. Many patients reported outcomes were measured, with limited consistent findings.
2020	Mavhu W, et al.	Effect of a differentiated service delivery model on virological failure in adolescents with HIV in Zimbabwe (Zvandiri): a cluster-randomised controlled trial	Quasi-experimental	Youth	Adherence	Adolescents with HIV at all clinics received adherence support through adult counsellors. At intervention clinics, adolescents with HIV were assigned a community adolescent treatment supporter, attended a monthly support group, and received text messages, calls, home visits, and clinic-based counselling. Implementation intensity was differentiated according to each adolescent’s HIV vulnerability, which was reassessed every 3 months. 496 adolescents, 212 were recruited at intervention sites and 284 at control sites. At 96 weeks, 52 (25%) of 209 adolescents in the intervention group and 97 (36%) of 270 adolescents in the control group had an HIV viral load of at least 1000 copies per μL or had died (adjusted prevalence ratio 0·58, 95% CI 0·36–0·94; p = 0·03).
*2020	Denison JA, et al.	Project YES! Youth Engaging for Success: A randomized controlled trial assessing the impact of a clinic-based peer mentoring program on viral suppression, adherence, and internalized stigma among HIV-positive youth (15–24 years) in Ndola, Zambia.	RCT	People living with HIV—youth	Community groups/clubs/ support	Randomized control trial (n = 273). Participants from the paediatric clinic experienced a relative increase in the odds of viral suppression by a factor of 4.7. There was no evidence of a study arm difference in viral suppression among youth in adult clinics or in ART adherence across clinic settings. Internalized stigma significantly reduced by a factor of 0.39 [OR:0.39, 95% CI:0.21, 0.73] in the intervention arm (50.4% to 25.4%) relative to the comparison arm (45.2% to 39.7%).
2021	Miller RL, et al.	Breaking down barriers to HIV care for gay and bisexual men and transgender women: The Advocacy and Other Tactics (ACT) Project	Prospective evaluation	Gay/bi men, trans women	Mobilization/ advocacy	Seven countries/collaborating partners. Investigators documented and verified 103 outcomes. Roughly two-thirds (n = 65; 63.1%) of the changes documented occurred to an individual or a small group of individuals and the remaining occurred in institutions (n = 38; 36.8%). The most common outcome was growth in consciousness and capability to ensure equal access to HIV treatment for gay and bisexual men and transgender women. The second most common category of outcomes resulting from the project was improvements in access to HIV care. The initiative led to increases in the coverage and framing of issues pertinent to accessing health care and human rights. The project also resulted in informal changes to exclusionary practices and norms. In a small number of instances (n = 3), outcomes occurred as formal policies. Although undesirable outcomes were also observed, these were a minority of outcomes (n = 9; 8.7%). Many of these undesirable consequences occurred for an individual or small group of individuals and concerned their loss of safety and security or access to resources.

* = Studies reporting mixed results or no differences in main outcomes measured between intervention and comparison arms.

### Study characteristics

While scarce, research on community-led responses and their outcomes appears to be gaining traction in recent years. Sixty-nine percent (n = 33) of articles included in this scoping review were published in the last 10 years between 2011 and early 2021. Fifty-six percent (n = 27) took place in the global south. South Africa (n = 4) and China (n = 3) were represented in the highest number of included studies from the global south [53–79]. The United States of America (n = 9) represented the highest number of studies that were implemented in the global north [80–94]. A diverse range of focus populations were represented in selected articles. Youth, gay and bisexual men, and people living with HIV were study populations in 27%, 23%, and 16% of articles included, respectively [56, 67, 69, 72, 75, 76, 78, 81, 84, 86, 88, 89, 94–99]. Research methods also varied. Twenty-seven percent of articles reported findings from quasi-experimental studies (n = 13) [54, 57, 58, 64, 67, 70, 72, 74, 77, 80, 81, 84, 86], 23% (n = 11) from systematic reviews [62, 63, 68, 69, 75, 83, 95, 96, 98–100], 19% (n = 9) from randomized control trials [55, 59, 61, 73, 76, 87–90], and 15% (n = 7) from prospective or longitudinal studies [56, 60, 65, 78, 79, 92, 93].

**Fig 2:**
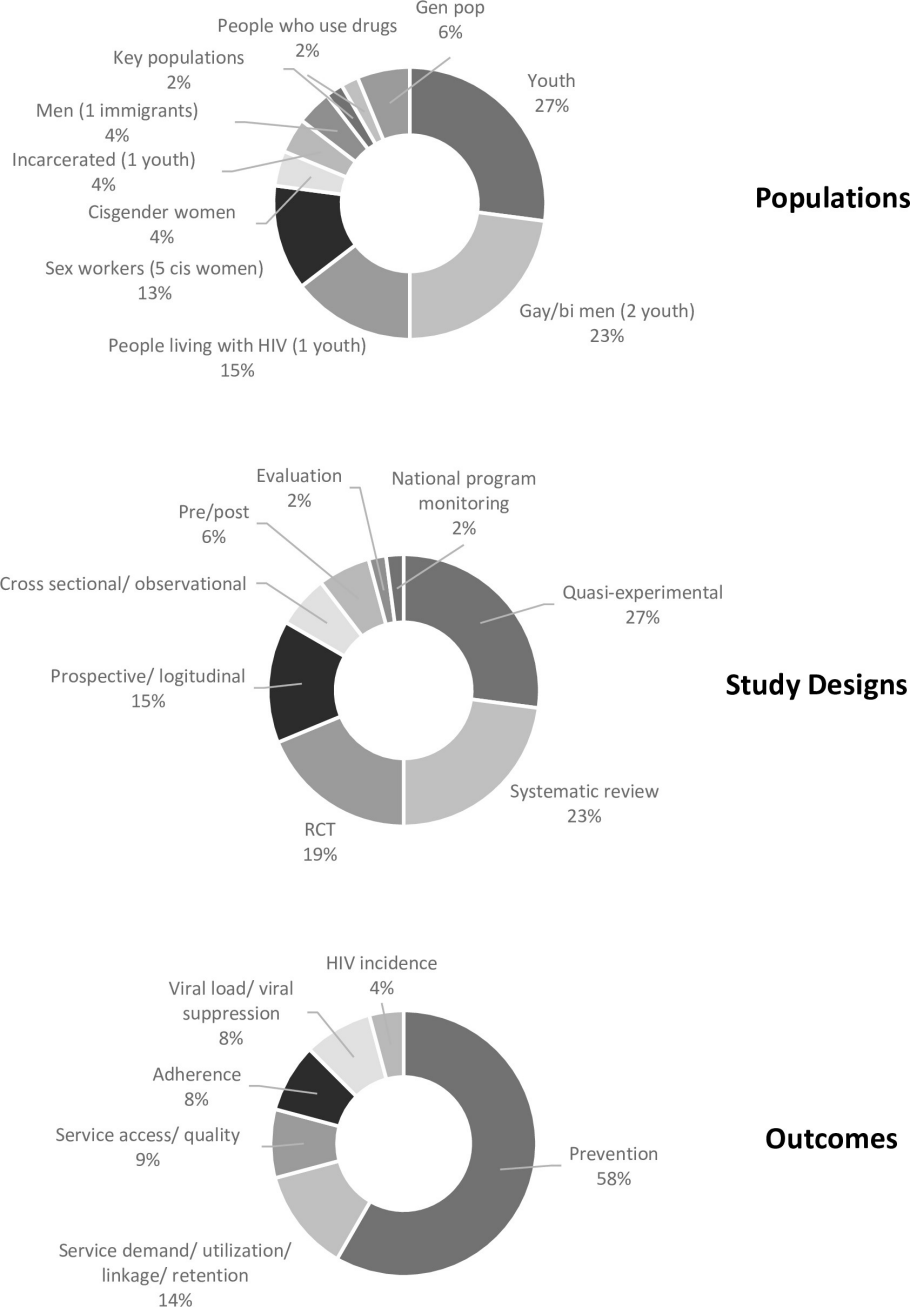
Study characteristics of research included in the scoping review.

A range of community-led approaches were described in the 48 articles we reviewed. Nearly half (48%, n = 23) described peer-led education or prevention interventions [54, 55, 57–62, 64–66, 70, 74, 79, 80, 82, 83, 85–88, 91, 96], of these more than half (n = 12) were focused on students or youth. Approaches in reviewed articles also include community-led testing, care, and treatment (n = 8) [56, 63, 67, 69, 73, 94, 97, 98], community mobilization, advocacy, monitoring, and human rights programs (n = 5) [53, 75, 78, 93, 100], community support groups, clubs, and mentors (n = 4) [71, 76, 89, 95], adherence programs (n = 4) [72, 77, 90, 99], community empowerment (n = 3) [68, 81, 84], and drop-in centres (n = 1) [92]. General characteristics of studies included in this scoping study are presented in Fig 2.

Outcomes studied also varied. At the individual level, more than half of studies (58%, n = 28) reported improved prevention outcomes, i.e., condom use, sexual risk, self-efficacy, attitudes, and intentions [53–55, 57–62, 64, 66, 70, 71, 74, 79–88, 91, 93, 96, 100]. There were 8 studies that reported improved HIV treatment adherence [89, 90, 95, 99] and viral load or viral suppression [56, 72, 76, 77]. Two studies report HIV incidence as an outcome [65, 68]. Findings that community-led responses led to improvements in HIV incidence were associative. At the service level, improvements were reported in 10 studies, including in the areas of access, quality, demand, linkage to care, utilization, community-provider relationships, and coordination [63, 67, 69, 73, 75, 78, 92, 94, 97, 98]. At the societal level, the beneficial effects of community-led HIV responses reported included: increases in community engagement, mobilization, social cohesion; and improvements in institutional norms and action planning [53, 75, 78, 93, 97, 100]. Community empowerment was reported as critically important for engaging sex workers and gay and bisexual men, although its benefit was implied for other populations as well.

There were 3 studies reporting the beneficial effects of community-led HIV responses at the structural level, 2 of which are systematic reviews. Outcomes reported in this category included broadened recognition of gay men and other men who have sex with men as a priority population, secured positive influence on policy, reduced stock outs of HIV-related commodities, increased adoption of viral load testing to monitor clinical outcomes, improved access to legal aid, increased awareness of rights on the part of both rights holders and duty bearers, and improved community-government relations [75, 78, 100]. A recent systematic review that examined human rights-related interventions, found improvements in HIV-related health outcomes in addition to positive changes at the structural-level. The same review also found a small number of interventions that had no or negative influence. These failures appeared to be related to incomplete initiatives, limited dissemination, or limited enforcement of study protocols (100).

Nine studies in our scoping review reported mixed results or no differences in main outcomes measured between intervention and comparison arms [60, 64, 69, 76, 86, 89, 90, 96, 99]. Efficacy seemed to vary by study design, with no improvements reported more often when analyses were restricted to randomized control trials. Reasons given by investigators for mixed efficacy results included risk of bias, misalignment between intervention design and intervention objectives, and failure to adequately assess both the contexts in which risk behaviours occur and intervention preferences among populations for which the studied intervention was intended.

Finally, two studies, each a systematic review, reported that community-led responses were cost effective or cost saving (i.e., per patient costs associated with HIV testing and counselling, health-services, adherence clubs, and costs associated with accessing services like transportation, childcare, lost work time) [63, 69]. Cost effectiveness is likely due to the adoption of community-led models with clinically stable patients, enabling communities to deliver care and treatment sustainably, cost-effectively, and equitably in resource-limited settings. Also contributing to cost effectiveness was the adoption of community-based or -led HIV testing and counselling approaches, which were found to be less expensive than facility-based strategies.

## Discussion

We found strong evidence to support expanded coverage of community-led HIV responses. Our scoping review revealed more than 40 beneficial outcomes linked to community-led HIV prevention, treatment, care, support, monitoring, and advocacy. More than half were prevention-related improvements. One prospective evaluation study of advocacy, conducted across 7 countries, documented, and verified 103 positive health and social inclusion outcomes over 24 months. Other investigators have similarly documented the critical importance of community engagement and the scale-up of peer-led prevention and treatment to fast-tracking the HIV response [2, 101–103].

We found study designs varied, with only 9 randomized control trials reported in the last 40 years. This finding makes sense given that randomized control trials may be exceedingly difficult to design and implement, given the multi-faceted and complementary nature of community-led HIV responses and the challenges inherent with meaningfully engaging key and vulnerable populations [104]. The absence of a previously agreed to definition has added to the complexity of studying community-led HIV responses. Outcomes measured also varied greatly, making it exceedingly difficult to draw comparisons between community-led approaches.

Most studies in our review took place in the global south and focused on peer-led approaches for students or youth. Five of the 15 studies that took place in the global north focused on gay and bisexual men. Studies focused specifically on people who use drugs, and transgender women represented a very small proportion of studies we examined, despite the potential benefits of community-led responses for these groups. For example, using a differentiated service delivery approach to prevention, testing, care, and treatment, delivered by and designed in consultation with men who have sex with men and transgender women, in partnership with the public health sector, can improve service coverage, reach, utilization, and retention [105].

Sixty percent of studies (n = 29) described more than one beneficial outcome linked to community-led HIV responses. This finding suggests that comprehensive community-led responses, especially when combined with structural level interventions, may have synergistic and simultaneous effects at more than just the individual level. This could be because programs were designed to address more than one outcome, or because when programs are community-led, clients’ needs are addressed holistically [106]. However, beneficial structural-level outcomes, e.g., changes in repressive laws and social attitudes, were rarely reported and were of mixed effectiveness [100]. This is not surprising given that societal and structural or legal changes operate on a longer time horizon than do traditionally measured public health outcomes and have multiple inputs, making advocacy programs more difficult to evaluate [107, 108].

Community-led HIV responses reported in the literature that we reviewed had several common characteristics that build on and reinforce the definition we used to conduct our scoping review. For example, some studies highlight the importance of empowerment and mobilization as effective strategies for engaging communities to lead HIV responses [41, 68, 93]. Relatedly, some studies underscore social cohesion as both an outcome and mediator of effective community-led HIV responses [93, 109]. Social networks might be another engine driving success. For example, understanding community-led HIV responses through a social networking lens, may shed light on how criminalized or stigmatized groups build power to influence change at the local level. This may be linked with the experience of affiliation, support, feeling valued, and making meaningful contributions to one’s community, each fundamental to social action and well-being [110–112]. Other researchers point to the importance of understanding community-led HIV responses as an iterative process that feeds back onto itself, beginning with constituency engagement, followed by alignment of adopted approaches with needs, adaptation of adopted approaches, and application of evidence gathered from monitoring and evaluation activities to influence policy changes [113] Also key are the inclusion of community-led responses in national AIDS plans and their funded operationalization at the local level. Bringing accountability closer to the level of service provision through community-led monitoring can increase the uptake and quality of HIV and other health services [69, 75, 78, 92, 97]. Moreover, sustained community activism for improved and sustained political commitment is vital for meeting HIV-related targets at local, national, regional, and global levels [114].

Together, the 48 studies we reviewed suggest comparative advantages of community-led HIV responses over facility-based, standard-of-care. Quantitative studies with comparison arms reinforce the importance of community-led prevention (i.e., HIV testing and counselling, risk reduction education and other behaviour change programs). Likewise, community-led components in treatment programs (i.e., adherence support, decentralized medication delivery) yield better service utilization as well as clinical outcomes. Our review also suggests that communities living with and disproportionately affected by HIV can effectively deliver services and influence policy. The comparative advantage of community-led HIV responses is predicated on several factors, including credibility with community members, ability to adapt to changing contexts and policy priorities, maintaining influence both within the community and at the policy level, community ownership, and iterative interactions and alliances with authorities resulting in accountability gains [31, 68, 115]. Likewise, several studies reiterated the point that having interventions that are community-based is insufficient for producing improved outcomes–interventions must be peer-led, of high quality, and possess strengthened capacity through skills training to ensure stronger, community-endorsed outcomes [57, 94, 116]. Peer-led responses are not only feasible but are also effective in producing higher service-related yields [117].

Formidable structural barriers to enabling community-led HIV response were repeatedly named in studies we reviewed. They include regressive laws and policies, funding constraints, and intersecting social stigmatizations, discrimination, and violence [68, 100]. Differentiated approaches to the delivery of HIV services might be a good bridge to enable expanded coverage of community-led HIV responses, especially in contexts that are hostile to key and vulnerable populations. This is because differentiated care flexibly tailors the provision of antiretroviral treatment for patients based on their acuity, greatly expanding the range of alternatives for how care occurs and who delivers it [72, 118–121].

At a time when funding for HIV is becoming more difficult given COVID-19’s detrimental impact and other competing priorities, the global HIV response needs to become more strategic in the investments it makes. Although research focused on community-led structural interventions is rare, studies we reviewed suggest that targeting social determinants shown in research to be associated with improved HIV outcomes—such as the availability of syringe programmes and comprehensive sexuality education, or removing barriers to high quality HIV and health services—have long been recognized as effective [63, 69, 75, 78, 100, 122]. Community empowerment and mobilization are also highly effective at engaging key and vulnerable populations, increasing service utilization and improving HIV-related health outcomes. They should become standard components of demand generation initiatives as well as testing, prevention, and violence mitigation programs [41, 68, 93, 123–126]. Additionally, we can become more strategic in combining community-led biomedical, behavioural, and structural interventions, and in so doing, leverage their synergistic effects [41]. Based on our scoping review and corroborated by other researchers, we should pursue better coverage of community-led, differentiated prevention, care, support, and treatment, socio-economic impact mitigation and other non-HIV support services [72, 118, 121]. Community-led services can be optimized when conducted in tactical and supportive partnerships with healthcare providers and government officials across health sectors [127–130]. Concurrently, some investment in high impact ‘disruptive innovations’ like HIV self-testing, multi-dose ARV dispensing for both prevention and treatment, adherence clubs, and drop-in centres may also be warranted. Disruptive innovations are interventions and program approaches that are inexpensive, rapid, consumer-controlled, and can be easily delivered in and by communities [73, 131].

### Limitations and strengths

There are a few important limitations to note. We restricted our scoping review to articles and reports published in English. Research published in other languages may have added to and/or validated the findings reported in this paper or might have contradicted them. Also, we used only three search engines–PubMed, Embase, and Web of Science–to conduct the article search. Other search engines may have yielded studies not included here. Finally, the limited number of published works reviewed in this scoping study, as well as the heterogeneity of research designs and outcomes reported, make it difficult to draw conclusions in many areas where community-led HIV responses might be beneficial. There is a need for more research to strengthen the evidence base undergirding normative guidance on the expanded role communities can play towards more effective and cost-efficient HIV responses. There is also a need for more studies showing the impact of community-led advocacy strategies focused on different issues across diverse contexts. In addition, research tools and protocols should be developed and made available to support community-led research in these areas.

Limitations notwithstanding, our scoping review allowed us to examine a broad and diverse range of research designs and outcomes [132]. This was especially important given the scarcity of research focused on community-led HIV responses. Our scoping review uncovered 9 probability-based randomized control trials, which is also worth noting. Although this study design is considered the gold standard for generalizability, such studies are costly and may be unethical to implement, especially in contexts that criminalize or stigmatize key and vulnerable populations. Creative study designs that are fit-for-purpose and can be community-led are warranted [133]. Indeed, sampling experts have advocated for innovative nonprobability sampling methods that are useful and cost-efficient, such as Internet sampling, especially in research with marginalized communities [134].

## Conclusions

Findings from this scoping review offer strong support for greater coverage of community-led HIV responses given their comparative advantages. To scale-up community-led HIV responses, we must first more meaningfully engage people living with HIV, key and vulnerable populations, and fund the organizations and networks they lead. In addition, we should:

1. Promote broad adoption of the definition of community-led HIV response included here, which can be applied uniformly across research, practice, and policy spheres. A universally accepted definition would make it easier to track investments, monitor effectiveness, and report results.
2. Implement prerequisite steps to establishing and supporting community-led HIV responses. They include strengthening technical and operational capacities of organizations led by people living with HIV, women, gay and bisexual men, people who use drugs, sex workers, transgender people, young people, and people with histories of incarceration. Special attention should be given to removing legal, policy, and funding barriers preventing community-led organizations from safely and efficiently operating [125, 130]. In addition, funding community empowerment and other processes that promote peer support and social cohesion among key and vulnerable populations may optimize the impact community-led responses can have [114, 135].
3. Curate prevention portfolios that are predominantly community-led and include two or more of the following: outreach; HIV testing–including self-testing; STI testing and treatment; comprehensive sexuality education; condom and lubricants; pre- and post-exposure prophylaxis (PrEP and PEP); behavioural interventions; harm reduction, including needle and syringe programmes; peer support; risk reduction counselling; and drop-in centres [136, 137]. Community-led prevention programs are especially important for driving down incidence curves among key and vulnerable populations [9].
4. Design treatment programs that have two or more well-funded, community-led components. Essential components include linkage to and coordination of care [67, 116]; decentralized dispensation of multi-dose ART that use differentiated care models to downstream treatment [72, 118, 121]; retention support [41, 69, 72]; adherence programs [56, 69, 72, 76, 95, 118]; home health [56, 138]; peer counselling and peer-led support groups [56, 139]; and treatment education [56]. At present, 27% of all people living with HIV worldwide are without treatment [140]. Our scoping review revealed evidence on the beneficial outcomes from community-led treatment, care, and support programmes, which when implemented with differentiated care models, can help to bridge the treatment gap [120].
5. Support community-led organizations that deliver services to empower and mobilize their clients/service recipients, monitor local HIV responses, advocate, expand access, mitigate and address violence, and generate demand for quality services [141]. Support for community-led monitoring and advocacy could also help ensure availability of medicines and diagnostics, while addressing service-related gaps and access barriers [75].
6. Leverage the synergistic effects of multi-component community-led responses that can amplify beneficial changes at individual, service, societal, and structural levels.(13) Also, invest in interventions that target multiple outcomes that are proximally related to HIV [131].
7. Conduct more research on community-led HIV responses, especially responses led by key and vulnerable populations. Research focused on programs led by people who use drugs and transgender people is especially needed. Studies are also needed on cost effectiveness of community-led HIV responses as well as on the long-term impact of structural-level interventions. Future research should adopt creative study designs and methods that are fit-for-purpose. For example, fractional factorial designs can identify independent and synergistic effects of intervention components and combination approaches [104, 142]. Communities of people living with HIV, key and vulnerable populations should be supported to lead research, including policy and evaluation studies [143–146]. Finally, the use of a consistent set of outcome measures focused on HIV and stronger integration of metrics used by health ministries, researchers, and program implementers should be encouraged. The need for more research should not preclude scaling of community-led responses.

The leadership of people living with and disproportionately affected by HIV is central to the global response. We must act rapidly to scale-up coverage of peer- and community-led programs and advocacy initiatives if we are to achieve the 2030 targets.

## Supporting information

S1 Checklist Preferred Reporting Items for Systematic reviews and Meta-Analyses extension for Scoping Reviews (PRISMA-ScR) checklist. (PDF)
